# Treatment optimization with concurrent SBRT and intracavitary brachytherapy for locally advanced cervical cancer

**DOI:** 10.1120/jacmp.v17i1.5610

**Published:** 2016-01-08

**Authors:** Bin Wan, Jinyi Lang, Pei Wang, C‐M Ma

**Affiliations:** ^1^ Department of Radiation Oncology Sichuan Cancer Hospital Chengdu Sichuan China; ^2^ Department of Radiation Oncology Fox Chase Cancer Center Philadelphia PA USA

**Keywords:** SBRT, intracavitary brachytherapy, treatment optimization, cervical cancer

## Abstract

This work is aimed at investigating treatment planning strategies to optimally combine stereotactic body radiation therapy (SBRT) with intracavitary brachytherapy (ICBT) for the treatment of locally advanced cervical cancer. Forty patients (stage IIB – IIIB) previously treated with combined SBRT and ICBT were randomly selected for this retrospective study. All patients were CT‐ and MR‐scanned with a ring applicator *in situ*. HR‐CTV and OARs were contoured according to fused CT and MR images. Several ICBT plans were generated for each patient based on different dose prescription points, and then a matching SBRT plan was generated for each ICBT plan. The dose distribution of each composite plan was analyzed with a focus on the doses received by 90% and 100% of the target volume ($D_{90}$ and $D_{100}$), the target volume receiving 100% of the prescription dose ($V_{100\%}$), and the doses received by 2 cc and 40% of the OARs ($D_{2\text{cc}}$ and $D_{40}$). As the distance, d, between the prescription point and the tandem varied within 1.0 and 1.9 cm, the $D_{90},\;D_{100}$ and $V_{100\%}$ for the target, as well as $D_{2\text{cc}}$ and $D_{40}$ for the bladder and rectum approached their optimal values for d value between 1.0 and 1.4 cm. When designing a combined ICBT+SBRT plan, one should measure the size of the cervix and set the prescription isodose line 1.0 to 1.4 cm away from the tandem for the ICBT plan first and then optimize the SBRT plan based on the ICBT dose distribution to achieve the best target coverage and critical structures sparing.

PACS number: 87.53.jw; 87.55.D‐

## I. INTRODUCTION

Brachytherapy, an important part of radiotherapy for cervical cancer, has garnered increasing interest in recent years. However, there are some obvious limitations associated with intracavitary brachytherapy (ICBT), including poor tumor coverage resulting from irregular or asymmetric target volumes. Previous investigations have reported two possible and favorable methods^1^, ^2^, ^3^); the alternative is to compensate the intracavitary dose distribution using a stereotactic body radiation therapy (SBRT) boost treatment (ICBT+SBRT). For extreme asymmetric tumors, it can be difficult to achieve optimal tumor coverage with ICBT+IS because of the challenges associated with placing needles in a desirable configuration.^2^, ^3^ On the other hand, when the tumor has a close relationship with an organ at risk (OAR), it can be difficult to achieve adequate tumor coverage by ICBT+SBRT due to dose constraints by the surrounding OARs.^(1)^ According to these investigations, whether ICBT+IS or ICBT+SBRT should be used for a particular patient depends on the size/shape of the cervix/tumor target and/or the relationship between the treatment volume and nearby OARs.

Dimopoulos et al.^(2)^ and Kirisits et al.^(3)^ have investigated the combination of ICBT+IS extensively. They developed a new applicator for large cervical tumors; namely, the Vienna Ring CT‐MR Applicator, which is based on the CT‐MR Ring Applicator, enhanced with the addition of nine guide holes in the ring tube, allowing placement of interstitial titanium needles using the ring tube as a needle template while maintaining the treatment channel of the ring tube. The addition of interstitial needles makes it possible to achieve asymmetric alteration of the dose distribution. The round‐point needle is designed to minimize tissue damage and improve ease in implantation. This applicator is designed to expand the target area without being limited by the OARs. However, the patients who accept the ICBT+IS treatment will have to endure a long treatment procedure, 5 to 8 hours from the implantation of needles to the treatment delivery,^(3)^ which is also a challenge for the nursing staff.

Regarding the PTV for brachytherapy for cervical cancer, Tanderup et al.^(4)^ strongly discouraged the application of PTV margins in the direction perpendicular to the uterine channel since this would lead to an overall dose escalation for the patient. This presents a significant challenge for conventional boost treatments using intensity‐modulated radiation therapy (IMRT) with conventional dose fractionation (e.g., 2 Gy/fraction) before or after the ICBT treatment since conventional IMRT typically requires a margin to account for the treatment planning and dose delivery uncertainties. Another concern relating to the combination of ICBT and conventional IMRT is the difficulty in calculating the overall biologically equivalent dose (BED) for the two modalities in various parts of the target volume and OARs because of the poor knowledge of the radiobiological response data for the tumor and related normal tissues under ICBT and IMRT dose/fractionation conditions. This problem can be solved by integrating ICBT with SBRT to deliver a combined dose distribution in conjunction with the same dose fractionation. A planning study by Assenholt et al.^(1)^ discussed the dosimetric characteristics of combined ICBT+SBRT plans.

In this work, we further investigated treatment planning strategies to achieve optimal dose distributions using combined ICBT+SBRT for advanced cervical cancers. We performed a retrospective dosimetric study on patients previously treated with combined ICBT+SBRT. We built the PTV with a small margin (<2 mm) based on a well‐controlled, image‐guided treatment procedure, in which the tumor target volume and relevant OARs were recontoured prior to each ICBT+SBRT treatment on CT and MR images with the applicator *in situ*. This ensures the consistency in anatomical geometry during ICBT+SBRT treatment planning and dose delivery. Several ICBT plans were generated for each patient case with different dose prescription points, and then a matching SBRT plan was generated for each ICBT plan to derive optimal combined ICBT+SBRT dose distributions, taking into account the tumor volume and shape and the target‐OARs relationship. Different dosimetric parameters were analyzed for these combined ICBT+SBRT plans to find suitable dose prescription points for the ICBT plan to integrate the two modalities most effectively.

## II. MATERIALS AND METHODS

### A. Patients and treatment history

Forty patients (22 stage IIB, 18 stage IIIB) previously treated with combined ICBT and SBRT (ICBT+SBRT) were randomly selected for this retrospective study. All patients received external beam radiation therapy (EBRT) to the whole pelvis (46 Gy in 23 fractions), followed by ICBT+SBRT (28 Gy in 4 fractions). Since the ICBT and SBRT doses were delivered consecutively in each fraction, their physical doses were summed up directly assuming the same biological effectiveness. The total equieffective prescription dose was 85 Gy (${\text{EQD}}_{2}$, assuming an α/β ratio of 10 Gy).

### B. Imaging for ICBT and SBRT

Fused CT and MR scans were used for structure contouring and dose calculation for all treatments. For each ICBT and SBRT treatment fraction, CT and MR scans were performed after the ring applicator implantation. The patient's bladder was emptied through a uterine catheter, and then injected with 50 ml to 120 ml normal saline (depending on the bladder volume of each patient) through the catheter before each image scan and treatment. The catheter valve was then shut off to ensure the same bladder volume during the entire imaging and treatment time. The range of the CT and MRI scans was between the L4‐L5 interspace to 3 cm in the vagina below the tumor.

### C. Target volume determination

The high‐risk target volume (HR‐CTV) and OARs (bladder, rectum, and sigmoid) were contoured according to the GEC‐ESTRO guidelines.^5^, ^6^, ^7^, ^8^ The HR‐CTV volumes in the 40 cases ranged from 70.36 cm^3^ to 188.71 cm^3^. The PTV for ICBT+SBRT treatment planning was built based on the HR‐CTV with a margin of 0.1 cm in the anterior–posterior direction and lateral directions, and 0.5 cm in the cranial direction.^(4)^

### D. Treatment planning details

For ICBT+SBRT treatment planning, an ICBT plan was first generated, and then a SBRT plan was optimized based on the dose distribution of the ICBT plan using the treatment goals and dose constraints for the target and OARs, as listed in Table 1. All plans were generated using the Oncentra MasterPlan System (version 4.3, SBRT accurate and final dose algorithm: collapsed cone, Elekta Medical Systems, Stockholm, Sweden).

**Figure 1:**
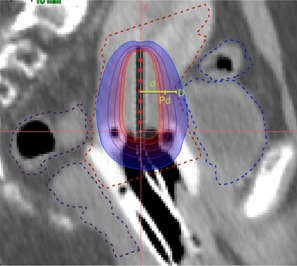
The sagittal view of a cervical cancer patient with a ring applicator *in situ*. The dose prescription point is $P_{d}$. D (in cm) is the distance between the outer wall of bladder and the center of the tandem and d (in cm) is the distance from point $P_{d}$ to the center of the tandem.

In order to investigate the optimal combination of the ICBT and SBRT dose distributions, several ICBT+SBRT plans were generated for each patient by choosing different dose prescription points, $P_{d}$, for the ICBT plans, which essentially set the ICBT dose proportion in the entire ICBT+SBRT plan. On the CT or MR images/fused CT‐MR scans, the slice that shows the central part of the tandem completely was selected in sagittal view. Assuming that D (cm) was the minimum distance from the outer wall of the target to the center of the tandem (Fig. 1) and $P_{d}$ was the dose prescription (7 Gy) point for the ICBT plan, the relative ICBT contribution to the entire ICBT+SBRT dose distribution varied, depending on D. According to the anatomy of all 40 patients investigated in this work, the D value varied significantly: 8 patients with D = 1.0 cm, 6 patients with D = 1.2 cm, 7 patients with D = 1.3 cm, 12 patients with D = 1.5 cm, 4 patients with D = 1.7 cm, and 3 patients with D = 1.8 cm.

**Table 1:** Optimization parameters for the target and OARs (bladder, rectum, and sigmoid) for ICBT+SBRT treatment planning.

*Name*	*Optimization Parameters*	*Type*	*Weight*
HR‐CTV	Min. dose 700 cGy, to 90% volume	Constraint	/
	Min. dose 650 cGy	Objective	150
Bladder	Max. dose 200 cGy, to 35% volume	Objective	100
	Max. dose 550 cGy	Constraint	/
Rectum	Max. dose 250 cGy, to 40% volume	Objective	100
	Max. dose 550 cGy	Constraint	/
Sigmoid	Max. dose 200 cGy, to 30% volume	Objective	100
	Max. dose 550 cGy	Constraint	/

For each patient with a given D, several ICBT plans were generated by setting the distance between $P_{d}$ and the center of the tandem, d, to different values (e.g., d = 0.5 cm, 0.6 cm, … D). Subsequently, a SBRT plan was generated based on the dose distribution of each ICBT plan. The SBRT plans used seven gantry angles including 180°, 129°, 78°, 27°, 333°, 290°, and 231°. The dose distributions of the four ICBT+SBRT fractions were combined based on deformable registration (Velocity Medical Solutions, Atlanta, GA). The final dose distributions were analyzed with a focus on the volume of HR‐CTV receiving 100% of the prescription dose ($V_{100\%}$), and the doses received by 2 cc and 40% of the OARs ($D_{2\text{cc}}$ and $D_{40}$).

### E. Plan evaluation

In order to evaluate the quality of each plan generated, we compared all plans with different d values for each patient based on the following parameters: the dose values received by 90% of the target volume ($D_{90}$), the dose received by 100% of the target volume ($D_{100}$), the volume of the HR‐CTV receiving 100% of the prescription dose ($V_{100\%}$), and the maximum doses received by 2 cc and 40% of the OARs ($D_{2\text{cc}}$ and $D_{40}$).

## III. RESULTS

**Figure 2:**
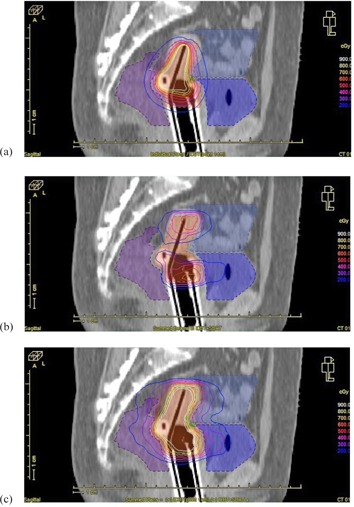
The relative dose distribution from ICBT only (a), the relative dose distribution from SBRT only (b), and total dose distribution for the combined ICBT+SBRT plan (c).

The treatment goal of the ICBT+SBRT technique was to treat the central portion of the target using ICBT with minimal effects on the surrounding OARs and to treat the peripheral regions of the target using SBRT to achieve the best target coverage and normal tissue sparing. Figure 2 shows the relative dose distribution from ICBT only, the relative dose distribution from SBRT only, and the combined dose distribution from both modalities. As seen clearly from the figure, the combined ICBT+SBRT treatment resulted in satisfactory target coverage and critical structure sparing.

**Figure 3:**
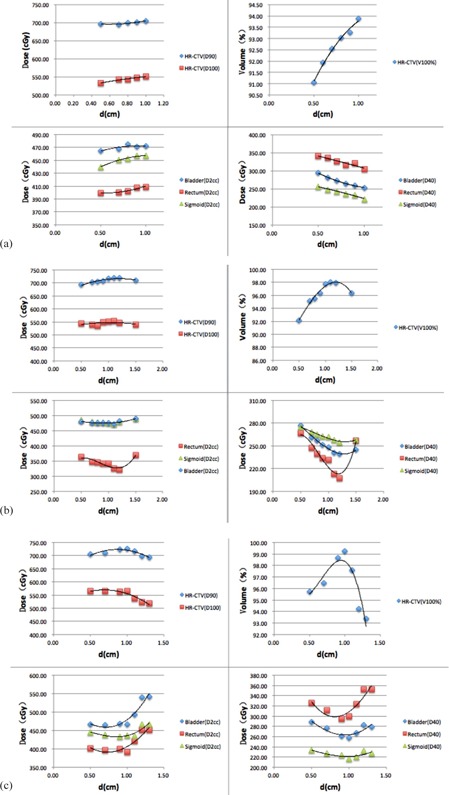
The variation of $D_{90},\;D_{100}$, and $V_{100\%}$ for the target and $D_{2\text{cc}}$ and $D_{40}$ for the OARs as a function of d: (a) a patient with D = 1.0 cm, (b) a patient with D = 1.3 cm, and (c) a patient with D = 1.5 cm. The trend lines are the best polynomial fits to the calculated data.

In this work, several ICBT+SBRT plans were generated for each patient based on different d values. Figure 3 shows the dose values received by 90% of the target volume ($D_{90}$), the dose received by 100% of the target volume ($D_{100}$), the volume of the HR‐CTV receiving 100% of the prescription dose ($V_{100\%}$), and the maximum doses received by 2 cc and 40% of the OARs ($D_{2\text{cc}}$ and $D_{40}$) as a function of d for three patients. The measured D values were 1.0 cm for Patient 1 (Fig. 3(a)), 1.3 cm for patient 2 (Fig. 3(b)), and 1.5 cm for patient 3 (Fig. 3(c)), respectively.

It is clear for the patient with D=1cm (Fig. 3(a)) that the $D_{40}$ values of the OARs approached minimal values when the $D_{90},\;D_{100}$, and $V_{100\%}$ values of the HR‐CTV reached their peaks, and the $D_{2\text{cc}}$ value reached its maximum value for d = D = 1.0 cm. The maximal $D_{2\text{cc}}$ values for the bladder, rectum, and sigmoid were 78.7%, 68.2%, and 76.2% of the prescription dose (increased by 1.6%, 2.3%, and 3.9% compared with the result for d = 0.5 cm), respectively, which were acceptable.^9^, ^10^ On the other hand, the $D_{90},\;D_{100}$, and $V_{100\%}$ values for the HR‐CTV increased by 1.3%, 3.4%, and 3.1%, and the $D_{40}$ values for the bladder, rectum, and sigmoid decreased by 14.0%, 10.4%, and 13.6% (compared with the result when d = 0.5 cm), respectively. From Fig. 3(b) and (c) it is evident that the $D_{2\text{cc}}$ and $D_{40}$ values of the OARs approached their minimal values when the $D_{90},\;D_{100}$, and $V_{100\%}$ values of the HR‐CTV reached their maximum.

**Figure 4:**
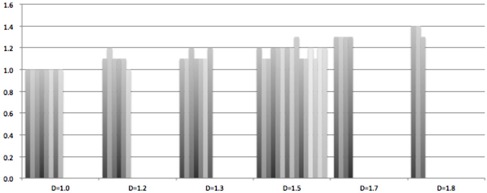
The optimal d value to achieve best $D_{90},\;D_{100}$, and $V_{100\%}$ of the HR‐CTV, and $D_{2\text{cc}}$ and $D_{40}$ of OARs for 40 patients with different D values.

From the results in Fig. 3 it can be seen that the $D_{90},\;D_{100}$, and $V_{100\%}$ values of the HR‐CTV, and $D_{2\text{cc}}$ and $D_{40}$ of the bladder and rectum vary with d and exhibit optimal values for a given D value. For the 40 patients investigated in this work, the optimal values of $D_{90},\;D_{100}$, and $V_{100\%}$ of the HR‐CTV, and $D_{2\text{cc}}$ and $D_{40}$ of OARs appeared at d = D for D = 1.0 cm (except for $D_{2\text{cc}}$ for critical structures), d approximately equal to 1.1 cm for D = 1.1 to 1.3 cm, d approximately equal to 1.2 cm for D = 1.5 cm, d approximately equal to 1.3 cm for D = 1.7 cm, and d approximately equal to 1.4 for D = 1.8 cm (see Fig. 4). The small variations among patients with identical D may be explained by the small differences in anatomical geometry and tumor volume/shape.

## IV. DISCUSSION

In this work, the equieffective prescription dose was 85 Gy at 2 Gy/fraction assuming an α/β ratio of 10 Gy for the target tissue (46 Gy external beam radiation therapy plus 4 fractions of ICBT+SBRT of 7 Gy/fraction). Compared with the results of Kirisits et al.^(9)^ and Assenholt et al.^(1)^ (based on Table 2), our findings were similar to that of the Kirisits study, but better than that of the work by Assenholt et al. It is also important to note that the average target volume in this work was 143.2% and 57.4% larger than that of Kirisits et al. and Assenholt et al., respectively.

It is clear based on the results summarized in Fig. 2 that optimal ICBT+SBRT plans can produce high‐quality dose distributions even for very large tumor volumes, and the doses to the OARs can be equally acceptable as ICBT+IS plans.^9^, ^10^ This demonstrates the feasibility of using ICBT+SBRT to achieve good dose coverage even when the tumor has an irregular shape, a large volume, and/or a close relationship with the OARs.

According to the analyses of the 40 cases investigated in this work, although patients may have different D values (e.g., different anatomical geometries), it is always possible to find an optimal combination of the ICBT and SBRT plans utilizing a suitable d value (e.g., an optimal dose prescription point) for the ICBT plan. This means that for a given D, there is an optimal way to combine the dose contributions of ICBT and SBRT that is also specific for the tumor volume/shape, which is determined by the dose prescription point for the ICBT plan. The optimal ICBT+SBRT plan with a well‐chosen dose prescription point (or optimal d) will have the maximal values of $D_{90},\;D_{100}$, and $V_{100\%}$ for the HR‐CTV and the minimal values of $D_{2\text{cc}}$ and $D_{40}$ for the OARs.

The above findings may be explained by several competing effects of ICBT and SBRT due to their radiation characteristics. ICBT is ideally suited for relatively small and symmetrical tumor volumes with minimal radiation damage to the surrounding OARs. The distance from the dose prescription point to the tandem (d) will increase as the tumor becomes larger, but the rapid dose falloff of ICBT also limits its application to finite tumor sizes. If the tumor volume is large or asymmetrical, it would be better to treat the central volume of the tumor (the core volume) with ICBT while treating the remaining peripheral tumor volume (the wrapper volume) with SBRT. However, SBRT dose distributions have their own limitations due to the characteristics of external radiation beams and the mechanical limits of the dose delivery system. For example, due to the step length and/or the leaf width of the multileaf collimator (MLC), the SBRT dose distributions are not suitable for very small wrapper volumes (e.g., very small in any directions), which sets a minimal wrapper volume limit for SBRT. Therefore, for relatively small tumor volumes with an asymmetrical shape, one may have to reduce the d value (i.e., to reduce the contribution of ICBT) in order to achieve the best match between the ICBT and SBRT dose distributions. For relatively large tumors with an asymmetrical shape, it would be reasonable to increase the d value to cover as much of the tumor core volume as possible until it reaches a certain limit. SBRT will then be utilized to cover the wrapper volume. As the SBRT contributions increase (i.e., for larger wrapper volumes), the doses to the surrounding OARs will increase because the external beams have to go through them. The optimal d value selection is a result of the balancing contributions from ICBT and SBRT in terms of the dosimetric parameters of interest (i.e., $D_{90},\;D_{100}$, and $V_{100\%}$ for the HR‐CTV and $D_{2\text{cc}}$ and $D_{40}$ for the OARs).

**Table 2:** Comparison of mean values of $D_{90},\;D_{100}$. and $V_{100}$ of the HR‐CTV, and $D_{2\text{cc}}$ and $D_{40}$ of the bladder and rectum between this work and previous publications.

	Mean Value±1 SD		
*Parameter*	*This work*	*Kirisits et al.* ^(9)^	*Assenholt et al.* ^(1)^
Prescription dose (α/β=10 Gy)	85 Gy	85±2 Gy	84 Gy
HR‐CTV (α/β=10 Gy)			
Volume	$107\pm 26\;{\text{cm}}^{3}$	$44\pm 27\;{\text{cm}}^{3}$	$68\pm 32\;{\text{cm}}^{3}$
$D_{100}$	70±8 Gy	70±6 Gy	69 Gy [68‐76]
$D_{90}$	94±10 Gy	96±12 Gy	87 Gy [79‐89]
$V_{100}$	95±6%	93±9%	96%[69%‐99%]
Bladder (α/β=3 Gy)			
$D_{2\text{cc}}$	84±11 Gy	83±14 Gy	87 Gy [59‐90]
$D_{40}$	46±4 Gy	NA	NA
Rectum (α/β=3 Gy)			
$D_{2\text{cc}}$	71±5 Gy	66±6 Gy	75 Gy [70‐75]
$D_{40}$	48±6 Gy	NA	NA
Sigmoid (α/β=3 Gy)			
$D_{2\text{cc}}$	69±11 Gy	67±7 Gy	72 Gy [66‐75]
$D_{40}$	45±5 Gy	NA	NA

For the bladder and rectum, for a small D (e.g., 1 cm or smaller), as the ICBT contribution increases (a larger core volume with an increasing d) the SBRT contribution will decrease accordingly (a smaller wrapper volume). The values of $D_{40}$ for the bladder and rectum will decrease because of less dose contributions from SBRT. However, due to the SBRT wrapper dose limitation, the increase of d will push the wrapper dose distribution outward, which will result in a small increase of $D_{2\text{cc}}$ for the bladder and rectum (Fig. 3(a)). For a larger D (e.g., 1.1 – 1.8 cm), the initial increase of ICBT dose contribution will help reduce $D_{2\text{cc}}$ and $D_{40}$ because of the reduced SBRT contribution. However, once ICBT reaches its physical limits, the $D_{2\text{cc}}$ and $D_{40}$ values for the bladder and rectum will begin to increase due to the increased dose contribution/spillover from SBRT (showing a turning point on the OAR curves in Fig. 3 (b) and (c)).

For the sigmoid, its volume and position can be affected by many factors, such as the shape of the uterus and the filling of the bladder. Fortunately the sigmoid will not receive high dose coverage at the same anatomy point, even for large interfractional movements. Therefore the sigmoid is not considered in this study.

For the target, if the cervix is small (e.g., for D<1 cm), one can increase the $D_{90},\;D_{100}$ and $V_{100\%}$ values of the HR‐CTV by increasing the contributions from the ICBT plan (e.g., the d value can be increased up to D). For larger D (e.g., 1.1 – 1.8 cm), we can increase the contributions from the ICBT plan to increase the target doses up to a certain limit. Beyond that limit, a further increase of d will push the SBRT wrapper dose distribution outward to cause a dose spillover that will increase the $D_{2\text{cc}}$ and $D_{40}$ values for the OARs. To compromise, the $D_{90},\;D_{100}$, and $V_{100\%}$ values of the HR‐CTV will decrease while the contribution of the ICBT plan further increases. It is interesting to see that the turning points of these dosimetric quantities appear at the same d values, demonstrating their competing effects on the ICBT and SBRT contributions to an optimal dose distribution for a specific anatomic geometry and tumor volume/shape.

## V. CONCLUSION

In this work, we have hypothesized that there is an optimal way to combine ICBT and SBRT plans to produce high‐quality dose distributions for large, irregular cervical cancers. A large number of test plans were generated to determine the optimal conditions for the two treatment modalities to form a perfect match that will result in optimal dosimetric parameters for the combined ICBT+SBRT treatments. We suggest a three‐step treatment planning procedure when designing an ICBT+SBRT plan. First, the D value will be measured according to the patient specific anatomy. Then, an appropriate d value (see Table 3) will be selected in order to normalize the 100% isodose line to this prescription dose point (the prescription dose is 7 Gy in this work). Finally, a SBRT plan will be optimized based on the ICBT plan. The final dose distribution of the combined ICBT+SBRT plan will generally meet the treatment requirement. One can adjust the prescription of the ICBT plan slightly, or equivalently to change the d value slightly, and then renormalize the SBRT plan accordingly to ensure one has obtained the best available ICBT+SBRT dose distribution for this patient.

In summary, the combination of ICBT with SBRT is superior to ICBT alone in that ICBT+SBRT provides improved dose coverage for large tumor volumes and asymmetric target shapes. In comparison with ICBT+IS, ICBT+SBRT also avoids the trauma and reduces the risks due to surgical procedures. Although ICBT+SBRT combines two different radiation modalities, the overall radiation treatment time is just between 15 to 20 minutes, and the total dose for the combined treatment can be simply calculated by summing up the doses from ICBT and SBRT directly.^5^, ^10^ The results from this work can be used to guide ICBT+SBRT treatment planning in the determination of dose prescription point for the ICBT plan and subsequent optimization of the SBRT plan to achieve optimal dose coverage to the treatment target while sparing critical structures effectively.

**Table 3:** The recommended d values as a function of D for combined ICBT+SBRT treatment planning.

D(cm)	≤1.0	1.0‐1.3	1.4‐1.5	1.6‐1.7	≥;1.8
d (cm)	D	1.1	1.2	1.3	1.4
